# High Burden of Undernutrition among At-Risk Children in Neonatal Follow-Up Clinic in Rwanda

**DOI:** 10.5334/aogh.2636

**Published:** 2020-10-01

**Authors:** Theoneste Mutsindashyaka, Alphonse Nshimyiryo, Kathryn Beck, Catherine M. Kirk, Kim Wilson, Christine Mutaganzwa, Jessica D. Bradford, Silas Havugarurema, Vianney Bihibindi, Patient K. Ngamije, Joel M. Mubiligi, Ann C. Miller

**Affiliations:** 1 Rwanda Ministry of Health, Kigali, RW; 2 Partners In Health/Inshuti Mu Buzima (PIH/IMB), Kigali, RW; 3 Boston Children’s Hospital, Boston, US; 4 Harvard Medical School, Boston, US

## Abstract

**Background:**

Sufficient knowledge of the disproportionate burden of undernutrition among vulnerable children is required for accelerating undernutrition reduction in low-income countries.

**Objectives:**

We aimed to assess the prevalence of stunting, underweight and wasting and associated factors among high-risk children born preterm, with low birth weight or other birth and neurodevelopmental injuries, who received nutritional support and clinical care follow-up in a Pediatric Development Clinic (PDC) in rural Rwanda.

**Methods:**

This cross-sectional study included all children from rural areas enrolled in PDC between April 2014–September 2017 aged 6–59 months at their last visit during this period. Anthropometric measurements, socioeconomic and clinical characteristics were extracted from an electronic medical records system. We used the World Health Organization child growth standards to classify stunting, underweight and wasting. Factors associated with undernutrition were identified using logistic regression analysis.

**Results:**

Of 641 children, 58.8% were stunted, 47.5% were underweight and 25.8% were wasted. Small for gestational age was associated with increased odds of stunting [OR 2.63; 95% CI 1.58–4.36] and underweight (OR 2.33; 95% CI 1.46–3.71), while history of feeding difficulties was significantly associated with wasting (OR: 3.36; 95% CI: 2.20–5.13) and underweight (OR: 2.68; 95% CI: 1.78–4.04). Later age at PDC enrollment was associated with increased odds of stunting (OR: 1.06; 95% CI: 1.01–1.11), underweight (OR: 1.09; 95% CI: 1.05–1.14) and wasting (OR: 1.07; 95% CI: 1.04–1.10).

**Conclusions:**

The prevalence of stunting, underweight and wasting are high in this at-risk population, highlighting the need for specific interventions to address undernutrition among children with similar characteristics. Early PDC enrollment of high-risk infants may reduce undernutrition risk.

## Introduction

Childhood undernutrition remains a global health challenge and more than 150 million children under five years of age, particularly in low- and middle-income countries (LMICs), are affected [1]. Stunting, low height-for-age, is a child’s failure to achieve full anticipated linear growth due to chronic undernutrition or poor health. Wasting, low weight-for-height, suggests a recent acute condition or shock leading to dropping weight, such as an illness or sudden lack of food. Underweight, low weight-for-age, can suggest either chronic or acute undernutrition. A heavy burden of undernutrition disproportionately affects children from rural communities, with lower socioeconomic status, and medical vulnerabilities such as prematurity, small for gestational age (SGA), low birth weight (LBW) or other birth and neurodevelopmental injuries and disabilities [2,3].

Despite global progress, malnutrition is especially concerning in sub-Saharan Africa (SSA). In 2016, one in three (36.7%) children under five years of age in Eastern Africa were stunted, while 6.5% were wasted [1]. Some children in SSA are at even higher risk for undernutrition due to perinatal risk factors. Prematurity (<37 weeks of gestation) is associated with almost double the odds of stunting, underweight and wasting compared to term babies [4]; LBW doubled the odds of stunting and wasting and tripled the odds of underweight for children compared to normal birthweight peers [4]. For children with birth injuries, such as asphyxia, the long term complications and disability can contribute to high risks of malnutrition in early childhood and beyond [5]. The burdens of prematurity and LBW are immense—affecting millions of children each year. It is estimated that 12% of all live births in SSA are preterm [6], 26% are SGA, and 2% face the double burden of preterm and SGA [7].

In Rwanda, childhood malnutrition is a significant problem—with 41% of children under five years stunted, 10% underweight and 2% wasted in rural areas [8]. While the burden of malnutrition in children with increased biologic or social vulnerability has not been fully documented in Rwanda, 78% of premature and/or LBW children discharged from a rural hospital neonatal unit were stunted and 9% wasted at ages 1–3 years [9].

Children with perinatal risk factors require long-term medical and nutritional follow-up for improved developmental outcomes [4,10,11]. In 2014, a Pediatric Development Clinic (PDC) was initiated by the Rwanda Ministry of Health (MOH), Partners In Health/Inshuti Mu Buzima (PIH/IMB) and UNICEF in eastern Rwanda [10]. The PDC provides a medical home model for medical, nutritional and developmental care of high-risk children up to age five after their discharge from specialized neonatal care services. Children are also referred later for specific high-risk conditions, such as developmental delay or post-central nervous system infections (cerebral malaria and meningitis).

The children served by PDC are highly vulnerable to undernutrition and developmental faltering [4,5,12]. A greater understanding of nutritional outcomes among children enrolled in PDC, particularly in SSA where malnutrition in the general population is high, is required for designing targeted interventions and accelerating the reduction of undernutrition in LMICs. This study aims to calculate the prevalence of stunting, underweight and wasting and assess associated factors among children who were enrolled in PDC.

## Methods

### Study Setting and Intervention

We conducted this study in the Rwinkwavu District Hospital (RDH) and Kirehe District Hospital (KDH) catchment areas in Kayonza and Kirehe districts, in eastern Rwanda. Both are Rwanda Ministry of Health operated facilities, which have been supported by PIH/IMB since 2005 and 2008, respectively. RDH supervises eight health centers in a catchment area of around 215,555 people and KDH supervises 16 health centers with a catchment area totaling 384,776 [13], in addition to a refugee camp of more than 50,000 people with two additional health centers.

PDC was initiated at RDH in April 2014 and later expanded to KDH in May 2016 with the aim to serve children born with prematurity, LBW (especially, children with a birthweight less than 2kg), hypoxic ischemic encephalopathy (HIE), hydrocephalus, cleft lip and palate, trisomy 21, or other developmental delays. Children could be referred to PDC from different hospital departments, including the neonatal care unit at time of discharge, health centers in the catchment area, or self-referred from the community. PDCs are run by a general nurse and social worker, with supervision by a general practitioner physician and technical support from PIH/IMB. Children are followed in the PDC until age five.

All children received a clinical, developmental and nutritional assessment at each visit and intervention plans based on the findings of the assessments. Nutritional interventions in PDC included group and individual nutrition-related counseling, food supplementation to children at risk for malnutrition and breast milk substitute, in cases when breastfeeding was not possible for infants under twelve months. Morning group education sessions focused on different topics, including introduction of complementary foods, dietary diversity, breastfeeding, hygiene, play and stimulation, and others. Children identified with uncomplicated severe or moderate wasting based on growth measurements were referred to the health center-based Outpatient Therapeutic or Supplementary Feeding Programs, respectively. More details about clinical and developmental services and PDC implementation can be found elsewhere [10].

### Study design and population

We conducted a cross-sectional study that included all children who had ever been enrolled in the PDC program between April 2014 and September 2017 and were aged 6–59 months (age adjusted for prematurity days when gestational age <37 weeks) at their last PDC visit during this period. The last visit was selected as the time of assessment to maximize each child’s exposure to the intervention in our study sample. Our study also included children with unknown gestational age, if reason for referral was not “preterm” and their age was between 6–59 months based on their birthdate and last visit date.

### Data Collection and Definition of Variables

#### Data collection

Data for this study were extracted from the OpenMRS [14] electronic medical record (EMR) and PDC patients’ charts. PDC providers record patients’ information on paper forms at enrollment and at every visit. Trained data collectors enter these data into EMR within one week of a visit. Additional data collected in patient charts, but not routinely entered into EMR, was collected for the study by trained data collectors and entered into EMR prior to data extraction.

#### Definition of variables

“Stunting”, “underweight” and “wasting” at last PDC visit were defined as length/height-for-age z-score < –2, weight-for-age z-score < –2 and weight-for-length/height z-score < –2, respectively, using World Health Organization (WHO) International Growth Standards [15].

“Child’s age at last visit” was adjusted for prematurity days when born <37 weeks. Adjusted age was defined as the child’s chronological age minus the number of days preterm (the difference in days between 40 weeks and the child’s gestational age). We grouped children into five age categories comparable to other research: 6–8 months, 9–11 months, 12–23 months, 24–35 months and 36–59 months [16,17].

“Small for gestational age (SGA)” was defined as birth weight less than 10th percentile for gestational age using the INTERGROWTH-21^st^ size at birth standards [18].

“Mutuelle de Santé” is Rwanda’s national community-based health insurance scheme and coverage was coded as yes or no [19].

“Ubudehe category” is a community-based ranking of households by socio-economic status in Rwanda [20,21]. Ubudehe has four categories with 1 being the poorest: Category 1 (i.e., families who do not own a house and can hardly afford basic needs), Category 2 (i.e., families who have a dwelling of their own or are able to rent one but rarely get full time jobs), Category 3 (i.e., families who have a job and farmers who go beyond subsistence farming to produce a surplus which can be sold) and Category 4 (i.e., families who own large-scale business, individuals working with international organizations and industries, and public servants). For this study, we combined Ubudehe category 3 and 4 as there were very few children in Ubudehe category 4.

“History of feeding difficulties” is defined as yes if a caregiver reported his or her child to have feeding difficulties at any visit at the PDC.

### Data Analysis

We described socio-demographic and clinical characteristics of children using frequencies and percentages for categorical data and median and interquartile ranges for continuous data. We conducted bivariate analysis using Chi-squared and Wilcoxon rank-sum tests to test association between each covariate and binary nutritional outcomes of stunted, underweight and wasted. Then, we used multivariable logistic regression models to identify factors associated with the outcomes, built using backward stepwise procedures for all variables significant at α = 0.10 in bivariate analyses. All factors significant at the α = 0.05 significance level were retained in the final model. We forced child’s sex and age at last visit in the final models for factors associated with stunting and underweight regardless of odds ratio results. The data were analyzed using Stata v.15.1 (Stata Corp, College Station, TX, USA).

### Ethics

The study received ethical approvals from the Rwanda National Ethics Committee and the Ministry of Health, and additional technical approvals from the PIH/IMB research committee, and the National Health Research Committee.

## Results

Of 641 children aged 6–59 months in our sample, 52.4% were boys and 73.9% were aged between 6–23 months (Table 1). The main reasons for referral to PDC are prematurity or LBW (45.4%), HIE (28.2%) and developmental delay (10.8%). Median chronological age at enrollment in PDC was younger for children enrolled in PDC for prematurity or LBW (1.05 months; IQR: 0.69–1.71) and HIE (0.89 months; IQR: 0.62–6.67), and was oldest for children with developmental delays (13.17 months; IQR: 8.41–24.57) (results not presented in tables). Of 583 children with data on feeding, 28.5% were reported to experience feeding difficulties. Feeding difficulties were highest among children with developmental delays (47.0%; n = 31/66) and lowest among preterm/LBW (19.3%; n = 52/270) (results not displayed in tables). In our sample, 58.8% of children were stunted, 47.5% were underweight and 25.8% were wasted. The burden of undernutrition (stunting: 62.1%, underweight: 62.7% and wasting: 45.4%) was highest among children enrolled in PDC for developmental delays. In addition, the prevalence of stunting (52.1%) and underweight (42.1%) were lowest among children with HIE, while the lowest prevalence of wasting (19.9%) was among children enrolled for prematurity or LBW.

**Table 1:** Associations between selected demographic and clinical characteristics of Pediatric Development Clinic (PDC) participants and their families with malnutrition outcomes; rural Rwanda, 2017^a^. n = 641 unless there were missing data.

Variable	Total	Stunted (n = 597)			Underweight (n = 619)			Wasted (n = 613)		

		No	Yes	p-value	No	Yes	p-value	No	Yes	p-value
		
	n (%)	n (%)	n (%)		n (%)	n (%)		n (%)	n (%)	

**All children**	**641 (100.0)**	**246 (41.2)**	**351 (58.8)**		**325 (52.5)**	**294 (47.5)**		**455 (74.2)**	**158 (25.8)**	
**Child’s sex**				0.056			0.024			0.405
Female	305 (47.6)	131 (45.3)	158 (54.7)		170 (57.2)	127 (42.8)		221 (76.0)	70 (24.0)	
Male	336 (52.4)	115 (37.3)	193 (62.7)		155 (48.1)	167 (51.9)		234 (72.7)	88 (27.3)	
**Child’s chronological age at enrollment in PDC (months), median [IQR]**	1.25 [0.72–7.36]	1.02 [0.66–2.53]	1.51[0.79–8.90]	<0.001	0.92[0.62–1.83]	1.97 [0.92–10.94]	<0.001	1.02 [0.66–2.83]	2.89 [1.05–14.13]	<0.001
**Child’s age at last PDC visit, corrected for prematurity days (in months)**				<0.001			0.002			0.007
6–8	121 (18.9)	66 (56.9)	50 (43.1)		69 (59.0)	48 (41.0)		84 (71.2)	34 (28.8)	
9–11	79 (12.3)	39 (51.3)	37 (48.7)		41 (52.6)	37 (47.4)		56 (72.7)	21 (27.3)	
12–23	274 (42.7)	102 (39.5)	156 (60.5)		149 (56.4)	115 (43.6)		205 (77.4)	60 (22.6)	
24–35	116 (18.1)	31 (30.1)	72 (69.9)		53 (47.3)	59 (52.7)		85 (80.2)	21 (19.8)	
36–59	51 (8.0)	8 (18.2)	36 (81.8)		13 (27.1)	35 (72.9)		25 (53.2)	22 (46.8)	
**Gestational age (weeks)**				0.529			0.836			0.103
≥37	172 (42.2)	67 (43.0)	89 (57.0)		99 (60.0)	66 (40.0)		123 (75.0)	41 (25.0)	
<37	236 (57.8)	102 (46.4)	118 (53.6)		134 (58.5)	95 (41.5)		186 (81.9)	41 (18.1)	
**Birth weight (kg)**				0.020			0.193			0.108
≥2.5	240 (42.5)	105 (46.9)	119 (53.1)		131 (57.5)	97 (42.5)		163 (71.5)	65 (28.5)	
<2.5	325 (57.5)	112 (36.6)	194 (63.4)		165 (51.7)	154 (48.3)		245 (77.8)	70 (22.2)	
**Small for gestational age (SGA)**				<0.001			<0.001			0.001
No	191 (29.8)	103 (58.5)	73 (41.5)		124 (68.1)	58 (31.9)		149 (82.3)	32 (17.7)	
Yes	180 (28.1)	49 (29.0)	120 (71.0)		90 (51.1)	86 (48.9)		134 (76.1)	42 (23.9)	
Unknown GA/birth weight	270 (41.1)	94 (37.3)	158 (62.7)		111 (42.5)	150 (57.5)		172 (67.2)	84 (32.8)	
**Relationship to primary caregiver**				0.214			0.166			0.753
Mother	489 (97.4)	182 (39.9)	274 (60.1)		243 (51.6)	228 (48.4)		344 (73.5)	124 (26.5)	
Other	13 (2.6)	2 (18.2)	9 (81.8)		4 (30.8)	9 (69.2)		9 (69.2)	4 (30.8)	
**Age of primary caregiver(years), median [IQR]**	27 [23–33]	27 [23–32]	27 [23–34]	0.363	26 [23–31]	28 [23–35]	0.007	27 [23–32]	30 [23–35]	0.028
**Caregiver marital status**				0.862			0.267			0.026
Married	408 (69.7)	153 (40.5)	225 (59.5)		214 (54.2)	181 (45.8)		301 (77.8)	86 (22.2)	
Living with partner (not married)	115 (19.7)	48 (42.9)	64 (57.1)		56 (50.0)	56 (50.0)		80 (70.2)	34 (29.8)	
Divorced/widowed	17 (2.9)	5 (38.5)	8 (67.5)		6 (42.9)	8 (57.1)		7 (46.7)	8 (53.3)	
Single	45 (7.7)	20 (46.5)	23 (53.5)		29 (65.9)	15 (34.1)		34 (79.1)	9 (20.9)	
**Caregiver years of schooling, median [IQR]**	5 [3–6]	5 [3–6]	5 [3–6]	0.874	5 [3–6]	5 [2–6]	0.136	5 [3–6]	5 [3–6]	0.659
**Mutuelle de Santé**				0.083			0.096			0.407
Yes	473 (84.8)	185 (41.9)	257 (58.1)		247 (54.3)	208 (45.7)		341 (75.8)	109 (24.2)	
No	85 (15.2)	25 (31.2)	55 (68.8)		37 (44.0)	47 (56.0)		59 (71.1)	24 (28.9)	
**Ubudehe category**				0.188			0.202			0.715
Category 1	27 (5.4)	6 (24.0)	19 (76.0)		9 (36.0)	16 (64.0)		18 (69.2)	8 (30.8)	
Category 2	379 (76.6)	138 (39.0)	216 (61.0)		189 (51.4)	179 (48.6)		271 (74.7)	92 (25.3)	
Category 3+	89 (18.0)	37 (44.6)	46 (55.4)		49 (56.3)	38 (43.7)		62 (72.1)	24 (27.9)	
**Total number of children/dependents in household, median [IQR]**	2 [1–4]	2 [1–4]	2 [1–4]	0.850	2 [1–4]	3 [1–5]	0.023	2 [1–4]	3 [1–5]	0.080
**Reason for referral: Preterm/low birth weight**				0.677			0.294			0.003
No	350 (54.6)	137 (42.0)	189 (58.0)		168 (50.4)	165 (49.6)		230 (69.3)	102 (30.7)	
Yes	291 (45.4)	109 (40.2)	162 (59.8)		157 (54.9)	129 (45.1)		225 (80.1)	56 (19.9)	
**Reason for referral: Hypoxic Ischemic Encephalopathy (HIE)**				0.042			0.106			0.412
No	460 (71.8)	165 (38.6)	263 (61.4)		226 (50.4)	222 (49.6)		323 (73.2)	118 (26.8)	
Yes	181 (28.2)	81 (47.9)	88 (52.1)		99 (57.9)	72 (42.1)		132 (76.7)	40 (23.3)	
**Reason for referral: Developmental delay**				0.598			0.009			<0.001
No	572 (89.2)	221 (41.6)	310 (58.4)		300 (54.3)	252 (45.7)		419 (76.6)	128 (23.4)	
Yes	69 (10.8)	25 (37.9)	41 (62.1)		25 (37.3)	42 (62.7)		36 (54.6)	30 (45.4)	
**Reason for referral: Other conditions**				0.808			0.192			0.173
No	555 (86.6)	214 (41.5)	302 (58.5)		288 (53.5)	250 (46.5)		400 (75.2)	132 (24.8)	
Yes	86 (13.4)	32 (39.5)	49 (60.5)		37 (45.7)	44 (54.3)		55 (67.9)	26 (32.1)	
**Reason for referral: Multiple conditions**				0.576			0.074			0.084
No	579 (90.3)	220 (40.8)	319 (59.2)		301 (53.8)	259 (46.2)		417 (75.3)	137 (24.7)	
Yes	62 (9.7)	26 (44.8)	32 (55.2)		24 (40.7)	35 (59.3)		38 (64.4)	21 (35.6)	
**Number of total visits in PDC, median [IQR]**	9 [5–13]	8 [5–12]	9 [5–13]	0.065	9 [6–13]	8 [5–12]	0.086	9 [6–13]	7 [5–11]	<0.001
**History of feeding difficulties**				0.103			<0.001			<0.001
No	417 (71.5)	166 (43.2)	218 (56.8)		242 (59.8)	163 (40.2)		325 (82.1)	71 (17.9)	
Yes	166 (28.5)	56 (35.4)	102 (64.6)		56 (35.0)	104 (65.0)		92 (56.4)	71 (43.6)	

^a^ All variables were evaluated at the child’s last PDC visit.

Factors associated with increased odds of stunting in the final model (Table 2), included SGA at birth (odds ratio (OR): 2.63; 95% confidence interval (CI): 1.58–4.36), not having mutuelle de santé (OR: 2.05; 95%CI: 1.14–3.66), being male (OR: 1.85; 95%CI: 1.23–2.79), having a low weight at birth (OR: 1.71; 95%CI: 1.06–2.75) and an increased age at enrollment in PDC (OR: 1.06; 95%CI: 1.01–1.11). In addition, there was increased odds of stunting among children with unknown gestational age or birth weight (OR: 2.25; 95%CI: 1.33–3.81) and a greater number of total visits in PDC (OR: 1.08; 95%CI: 1.01–1.15).

**Table 2:** Results of the multivariable analysis of factors associated with stunting in Pediatric Development Clinic (PDC) participants, Rwanda, 2017.

Variable	Full model		Final model	

	OR [95%CI]	p-value	OR [95%CI]	p-value

**Child’s sex**				
Female	1.00		1.00	
Male	1.69 [1.10–2.60]	0.016	1.85 [1.23–2.79]	0.003
**Child’s chronological age at enrollment in PDC (months)**	1.06 [1.00–1.11]	0.034	1.06 [1.01–1.11]	0.016
**Child’s age at last PDC visit, corrected for prematurity days (in months)**				
6–8	1.00		1.00	
9–11	1.14 [0.54–2.44]	0.726	1.35 [0.66–2.77]	0.416
12–23	1.36 [0.71–2.59]	0.350	1.45 [0.79–2.66]	0.236
24–35	1.87 [0.72–4.87]	0.197	1.75 [0.71–4.31]	0.224
36–59	1.62 [0.29–9.16]	0.583	1.23 [0.26–5.78]	0.790
**Birth weight (kg)**				
≥2.5	1.00		1.00	
<2.5	2.12 [1.14–3.96]	0.018	1.71 [1.06–2.75]	0.027
**Small for gestational age (SGA)**				
No	1.00		1.00	
Yes	2.84 [1.68–4.82]	< 0.001	2.63 [1.58–4.36]	< 0.001
Unknown gestational age/birth weight	2.36 [1.35–4.12]	0.003	2.25 [1.33–3.81]	0.003
**Mutuelle de Santé**				
Yes	1.00		1.00	
No	1.71 [0.91–3.21]	0.093	2.05 [1.14–3.66]	0.016
**Reason for referral: Hypoxic Ischemic Encephalopathy (HIE)**				
No	1.00			
Yes	1.16 [0.63–2.13]	0.641		
**Number of total visits in PDC**	1.08 [1.01–1.15]	0.029	1.08 [1.01–1.15]	0.017
**History of feeding difficulties**				
No	1.00			
Yes	1.36 [0.83–2.24]	0.222		

Factors associated with increased odds of underweight in the final model (Table 3) included history of feeding difficulties (OR: 2.68; 95%CI: 1.78–4.04); SGA at birth (OR: 2.33; 95%CI: 1.46–3.71) and later enrollment in PDC (OR: 1.09; 95%CI: 1.05–1.14). Increased odds of underweight were also observed in children with unknown gestational or birth weight (OR: 1.90; 95%CI: 1.20–3.02) and a greater number of total PDC visits (OR: 1.06; 95%CI: 1.01–1.11).

**Table 3:** Results of the multivariable analysis of factors associated with underweight in Pediatric Development Clinic (PDC) participants, Rwanda, 2017.

Variable	Full model		Final model	

	OR [95%CI]	p-value	OR [95%CI]	p-value

**Child’s sex**				
Female	1.00		1.00	
Male	1.53 [1.02–2.30]	0.041	1.39 [0.97–1.99]	0.073
**Child’s chronological age at enrollment in PDC (months)**	1.10 [1.05–1.15]	<0.001	1.09 [1.05–1.14]	<0.001
**Child’s age at last PDC visit, corrected for prematurity days (in months)**				
6–8	1.00		1.00	
9–11	0.95 [0.45–2.01]	0.902	0.96 [0.51–1.82]	0.903
12–23	0.72 [0.37–1.38]	0.318	0.70 [0.40–1.21]	0.201
24–35	0.70 [0.28–1.75]	0.450	0.65 [0.30–1.42]	0.282
36–59	0.19 [0.04–0.99]	0.049	0.29 [0.07–1.16]	0.080
**Small for gestational age (SGA)**				
No	1.00		1.00	
Yes	2.13 [1.25–3.61]	0.005	2.33 [1.46–3.71]	<0.001
Unknown gestational age/birth weight	1.85 [1.07–3.22]	0.028	1.90 [1.20–3.02]	0.006
**Age of primary caregiver(years)**	1.02 [0.98–1.05]	0.395		
**Mutuelle de Santé**				
Yes	1.00			
No	1.43 [0.79–2.57]	0.237		
**Total number of children/dependents in Household**	0.98 [0.86–1.11]	0.698		
**Reason for referral: Developmental delay**				
No	1.00			
Yes	0.71 [0.30–1.66]	0.432		
**Reason for referral: Multiple conditions**				
No	1.00			
Yes	1.07 [0.46–2.48]	0.872		
**Number of total visit in PDC**	1.07 [1.01–1.14]	0.021	1.06 [1.01–1.11]	0.030
**History of feeding difficulties**				
No	1.00		1.00	
Yes	2.92 [1.81–4.70]	<0.001	2.68 [1.78–4.04]	<0.001

History of feeding difficulties (OR: 3.36; 95%CI: 2.20–5.13) and later enrollment in PDC (OR: 1.07; 95%CI: 1.04–1.10) were significantly associated with increased odds of wasting (Table 4). The odds of wasting were significantly lower among older children compared to children aged 6–8 months: 12–23 months (OR: 0.55; 95%CI: 0.32–0.96), 24–35 months (OR: 0.35; 95%CI: 0.16–0.76) and 36–59 months (OR: 0.25; 95%CI: 0.08–0.78).

**Table 4:** Results of the multivariable analysis of factors associated with wasting in Pediatric Development Clinic (PDC) participants, Rwanda, 2017.

Variable	Full model		Final model	

	OR [95%CI]	p-value	OR [95%CI]	p-value

**Child’s chronological age at enrollment in PDC (months)**	1.13 [1.03–1.23]	0.009	1.07 [1.04–1.10]	<0.001
**Child’s age at last PDC visit, corrected for prematurity days (in months)**				
6–8	1.00		1.00	
9–11	0.58 [0.20–1.66]	0.312	0.69 [0.34–1.40]	0.304
12–23	0.27 [0.10–0.73]	0.010	0.55 [0.32–0.96]	0.034
24–35	0.15 [0.02–0.79]	0.025	0.35 [0.16–0.76]	0.007
36–59	0.50 [0.03–7.93]	0.620	0.25 [0.08–0.78]	0.017
**Gestational age (weeks)**				
≥ 37	1.00			
< 37	0.60 [0.25–1.43]	0.251		
**Small for gestational age (SGA)**				
No	1.00			
Yes	1.31 [0.63–2.69]	0.468		
Unknown gestational age/birth weight	0.87 [0.25–3.10]	0.836		
**Age of primary caregiver(years)**	0.98 [0.92–1.04]	0.444		
**Caregiver marital status**				
Married	1.00			
Living with partner (not married)	0.66 [0.27–1.60]	0.360		
Divorced/widowed	3.06 [0.35–26.58]	0.311		
Single	0.65 [0.15–2.73]	0.552		
**Total number of children/dependents in Household**	1.17 [0.99–1.39]	0.071		
**Reason for referral: Preterm/low birth weight**				
No	1.00			
Yes	1.56 [0.59–4.11]	0.373		
**Reason for referral: Developmental delay**				
No	1.00			
Yes	3.60 [0.58–22.23]	0.168		
**Reason for referral: Multiple conditions**				
No	1.00			
Yes	0.65 [0.14–3.04]	0.581		
**Number of total visits in PDC**	1.03 [0.93–1.14]	0.567		
**History of feeding difficulties**				
No	1.00		1.00	
Yes	2.78 [1.37–5.62]	0.004	3.36 [2.20–5.13]	<0.001

## Discussion

We found high prevalence of stunting, underweight and wasting among children aged 6–59 months who received nutrition, development and medical follow-up in PDC between 2014 and 2017. The odds of wasting were particularly high among younger children and SGA was associated with increased odds of stunting and underweight. Importantly, early PDC intervention was associated with reduced odds of stunting, underweight and wasting.

As expected, there was higher burden of stunting, underweight, and wasting (58.8%, 47.5%, 25.8%, respectively) among children enrolled in the PDC aged 6–59 months between 2014 and 2017, compared to the national prevalence of undernutrition among children under five years of age in rural Rwanda (stunting: 40.6%, underweight: 10.0% and wasting: 2.3%) [8]. Two earlier studies in Rwanda and Burundi showed elevated rates of stunting, underweight and wasting among preterm and LBW infants, however stunting in our sample was lower (58.8% in PDC versus 79.0% in Rwanda without PDC at ages 12–36 months and 81.0% in Burundi at age 2 years) and prevalence of wasting was higher (25.8% PDC versus 9.0% and 18.0%, respectively) [9,22]. It is important to note that both of these studies had a large number of children that could not be located for assessment (23–46%) or died (4–6%) indicating potential survival bias which might explain the lower overall wasting prevalence given that the children lost to follow-up had characteristics that put them at high risk for wasting and death [23].

The burden of undernutrition has declined globally [1], but our study highlights a heavy burden of undernutrition among high-risk children that current health services are not adequately addressing. The PDC population, consisting primarily of children born preterm/LBW and with neurodevelopmental injuries, are at high risk of malnutrition [23], but with comprehensive follow-up in the PDC, there appear to be lower rates of malnutrition compared to other studies where there was no follow-up, particularly for stunting. Furthermore, while children in PDC receive additional nutritional services, the high rates of undernutrition we report in this population indicate that even more specialized services are needed for the highest risk children beyond what is currently provided within the PDC. For instance, children with feeding difficulties require specialized interventions [24] that may be beyond the scope of management by general nurses and social workers in the PDC. Quality improvement efforts are on-going to strengthen PDC nutritional screening and intervention [25]. This study highlights a tremendous burden of malnutrition among children with feeding difficulties and developmental delay. Other research has highlighted feeding difficulties and subsequent malnutrition to be more prevalent among children with cerebral palsy and other disabilities [5,26,27,28,29], which are an underserved group of children in LMICs.

Our findings indicate that younger children have higher odds of wasting, which was consistently found by other studies in SSA [30]. The highest risk group (6–8 months) are children who are in the age range when transition from exclusive breastfeeding to complementary feeding should take place [17]. This might be due to inappropriate or non-exclusive breastfeeding among children under six months, delayed introduction of complementary feeding, inappropriate complementary feeding and children with feeding difficulties or disability [31,32]. Interventions, such as caregiver education and counseling on exclusive breastfeeding under six months, maternal nutrition among lactating women, timely transition to and adequate complementary feeding should be considered in PDC in addition to addressing potential underlying risk factors for sub-optimal feeding such as food insecurity. It is also possible that some of the lower rates of wasting at older ages may be due to lower levels of survival among children with wasting at younger ages, given the link between wasting and increased mortality risk [33].

We found that SGA is an important risk factor for chronic malnutrition (stunting and underweight) in our study. This may indicate that children born SGA do not “catch up” on the growth restriction that occurred in utero, even in the presence of a follow-up clinic. This is supported by global evidence of intrauterine growth restriction, or small size at birth, being a major contributor to future growth faltering [34,35]. To address this, interventions to complement PDC must focus on the life cycle and address factors that contribute to growth restriction in pregnancy, such as timely and high-quality antenatal care, birth spacing, reducing poverty, and improved water and sanitation and hygiene [34,35].

Early age of enrollment in PDC was a protective factor for stunting, underweight and wasting. This finding is consistent with other studies which show that early intervention improves developmental outcomes of at-risk children [11]. This provides promising evidence that PDC is helping to address the challenge of malnutrition among this high-risk population of children when they are enrolled soon after birth. The PDC model aims to refer the majority of infants to PDC one week after their discharge from the hospital. Additional efforts should be made to ensure these guidelines are followed to allow for early intervention services that also ensure appropriate transition to complementary feeding, and strengthening services around nutrition to support the population of children with feeding difficulties and developmental delays for whom current interventions are insufficient given the high rates of malnutrition found in this study.

Our study has limitations. First, as we used routinely collected data for PDC, missing data was a limitation (birth weight and gestational age, for example, were not always available). Additionally, history of dietary intake of children aged 6–59 months, is not routinely documented on PDC visit forms, and so these data were not available. However, we collected data on caregivers’ reporting the child having any feeding difficulties to provide some context. Second, because all of the children in this study were enrolled in PDC, these estimates of undernutrition indicators may not be generalizable to a population not receiving the same supportive services. However, despite these limitations, we still believe the information is valuable to inform the need for intervention, as we believe that this was the first effort in Rwanda to quantify malnutrition in this high-risk population of children 6–59 months of age.

In conclusion, the prevalence of stunting, underweight and wasting are high in this PDC population of children born preterm, LBW, with HIE or experiencing other neurodevelopmental difficulties at age 6–59 months, although these children had exposure to the PDC’s nutrition, developmental, and medical support. Early enrollment in PDC of high-risk infants may reduce risk of undernutrition. However, some prenatal and perinatal factors that are not able to be addressed by PDC, such as SGA, increase odds of undernutrition and require interventions to prevent intrauterine growth restriction, HIE, and prematurity. Further, our findings highlight the need for specialized focus among children with feeding difficulties and developmental delay both in the PDC and in other sub-Sahara African countries where services are limited or not available.
